# Adenosine A_2A_ Receptors in the Rat Prelimbic Medial Prefrontal Cortex Control Delay-Based Cost-Benefit Decision Making

**DOI:** 10.3389/fnmol.2018.00475

**Published:** 2018-12-20

**Authors:** Douglas T. Leffa, Pablo Pandolfo, Nélio Gonçalves, Nuno J. Machado, Carolina M. de Souza, Joana I. Real, António C. Silva, Henrique B. Silva, Attila Köfalvi, Rodrigo A. Cunha, Samira G. Ferreira

**Affiliations:** ^1^CNC-Center for Neuroscience and Cell Biology, University of Coimbra, Coimbra, Portugal; ^2^Department of Biochemistry, Federal University of Rio Grande do Sul, Porto Alegre, Brazil; ^3^Department of Neurobiology, Fluminense Federal University, Niterói, Brazil; ^4^Post-Graduate Program in Medical Sciences, Faculty of Medicine, Federal University of Ceará, Fortaleza, Brazil; ^5^Faculty of Medicine, University of Coimbra, Coimbra, Portugal

**Keywords:** adenosine A_2A_ receptors, impulsive choice, prefrontal cortex (PFC), anxiety, working memory, cost-benefit decision making

## Abstract

Adenosine A_2A_ receptors (A_2A_Rs) were recently described to control synaptic plasticity and network activity in the prefrontal cortex (PFC). We now probed the role of these PFC A_2A_R by evaluating the behavioral performance (locomotor activity, anxiety-related behavior, cost-benefit decision making and working memory) of rats upon downregulation of A_2A_R selectively in the prelimbic medial PFC (PLmPFC) via viral small hairpin RNA targeting the A_2A_R (shA_2A_R). The most evident alteration observed in shA_2A_R-treated rats, when compared to sh-control (shCTRL)-treated rats, was a decrease in the choice of the large reward upon an imposed delay of 15 s assessed in a T-maze-based cost-benefit decision-making paradigm, suggestive of impulsive decision making. Spontaneous locomotion in the open field was not altered, suggesting no changes in exploratory behavior. Furthermore, rats treated with shA_2A_R in the PLmPFC also displayed a tendency for higher anxiety levels in the elevated plus maze (less entries in the open arms), but not in the open field test (time spent in the center was not affected). Finally, working memory performance was not significantly altered, as revealed by the spontaneous alternation in the Y-maze test and the latency to reach the platform in the repeated trial Morris water maze. These findings constitute the first direct demonstration of a role of PFC A_2A_R in the control of behavior in physiological conditions, showing their major contribution for the control of delay-based cost-benefit decisions.

## Introduction

Adenosine A_2A_ receptors (A_2A_Rs) are mostly known to control long-term synaptic plasticity throughout the brain (reviewed in Cunha, 2016), namely in the prefrontal cortex (PFC) where they facilitate long-term potentiation (LTP) in excitatory synapses onto fast spiking interneurons and control network activity (Kerkhofs et al., 2018). The PFC mediates cognitive and executive functions including working memory, attention and inhibitory control (Goldman-Rakic, 1999; Fuster, 2001), which are disrupted in major neuropsychiatric disorders such as attention deficit and hyperactivity disorder (ADHD), addiction and schizophrenia (Arnsten et al., 2015). Notably, the antagonism of A_2A_R has been tied to the improvement of mood and memory deficits in several neuropsychiatric disorders (Chen, 2014; Kaster et al., 2015; Viana da Silva et al., 2016). The contribution of A_2A_R in the PFC is suggested by the observation that the antagonism of adenosine receptors with their general antagonist caffeine improves attention and short-term memory in animal models of ADHD (Caballero et al., 2011; Pandolfo et al., 2013). Furthermore, A_2A_R antagonism increases impulsivity (Oliveros et al., 2017), attenuate the effects of dopamine D_2_ receptor antagonism on effort-based decision making (Pardo et al., 2012) and attenuate working memory deficits in rats with PFC dopamine depletion (Horita et al., 2013).

While optogenetic activation of A_2A_R signaling pathways in the medial PFC improves maintenance of spatial working memory (Li et al., 2018), there are still no direct evidence supporting a role for the endogenous activation of A_2A_R in the PFC to modulate behavior. This is of particular relevance since A_2A_R are present in different areas of the forebrain (i.e., cerebral cortex, hippocampus and striatum) with different impacts on different behavioral outputs, as heralded by the striking opposite phenotypes resulting from the selective deletion of A_2A_R from only the striatum or forebrain neurons (Shen et al., 2008, 2013; Wei et al., 2014). Thus, to better understand the role of the endogenous activation of A_2A_R in the PFC, we now selectively downregulated A_2A_R in the rat prelimbic medial PFC (PLmPFC) and evaluated the consequences on PFC-related behaviors such as working memory, anxiety-related behavior and delay-based cost-benefit decision-making. Our findings reveal that the downregulation of A_2A_R in the PLmPFC decreased the choice of the large reward in a T-maze-based cost-benefit paradigm in which the cost was delay, suggesting an increase in impulsive decision making, a finding relevant for disorders with impaired decision making, such as Parkinson’s disease, schizophrenia, ADHD and addiction (Lee, 2013).

## Materials and Methods

### Animals

Male Wistar rats (7-week-old) were purchased from Charles River (Barcelona, Spain) and housed in a temperature and humidity-controlled environment with 12 h light on/off cycles and *ad libitum* access to food and water. All studies were conducted in accordance with the principles and procedures outlined as “3Rs” in the EU guidelines (210/63), FELASA, and the National Centre for the 3Rs (the ARRIVE; Kilkenny et al., 2010), and were approved by the Animal Care Committee of the Center for Neuroscience and Cell Biology (ORBEA 78/2013).

### Generation and Bilateral Administration of Lentiviral Vectors Into the PLmPFC

A small hairpin RNA targeting A_2A_R (shA_2A_R, nt 419–437) was inserted into a lentivector together with an enhanced green fluorescent protein (EGFP) reporter gene, as previously detailed (Simões et al., 2016; Viana da Silva et al., 2016). This shA_2A_R has been shown to cause a 68% decrease of A_2A_R mRNA expression and a 55% decrease of A_2A_R protein density in the striatum, where the high density of A_2A_R allows a faithful quantification (Viana da Silva et al., 2016). A hairpin targeting the coding region of red fluorescent protein (nt 22–41) was used as an internal control (shCTRL). These lentivectors (1 μL per hemisphere at 750,000 ng of p24 antigen/mL) were stereotaxically delivered into the PLmPFC of the two hemispheres at an infusion rate of 0.2 μL/min in the following coordinates: antero-posterior: +3.20 mm; lateral: ±0.60 mm; dorso-ventral: −3.80 mm (Paxinos and Watson, 2009).

### Radioligand Binding Assay in Total Membranes From the PLmPFC

The amount of tissue allowed a single point radioligand binding which was carried out with slight modification to our previous studies (Cunha et al., 1999; Ferreira et al., 2015). Three male Wistar rats of 6–8 weeks of age were bilaterally injected shA_2A_R in their PLmPFC, while four Wistar rats were bilaterally injected with the shCTRL. At 5 weeks post-injection, rats were decapitated under halothane anesthesia, and their brains transferred to ice-cold artificial cerebrospinal fluid (composition in mM: NaCl 125, KCl 3, MgSO_4_ 1, CaCl_2_ 2, Na_2_HPO_4_ 1.25, NaHCO_3_ 25–26 and glucose 11, pH 7.4 (osmolality of 300 mOsmol/kg), oxygenated with carbogen (95% O_2_ + 5% CO_2_). We obtained coronal brain slices from which we dissected the PLmPFC, which was homogenized in 1.8 mL of ice-cold membrane preparation solution of the following composition: sucrose (320 mM), EDTA (2 mM), MgCl_2_ (3 mM), HEPES (15 mM), pH 7.4, supplied with a protease inhibitor cocktail (Sigma-Aldrich, 1 μL/mL). The homogenates were then centrifuged at 1,000 *g* for 30 min, at 4°C to decant intracellular debris. The membrane-rich supernatant was then re-centrifuged at 20,000 *g* for 30 min, and the pellets were vigorously resuspended in 450 μL binding assay buffer of the following composition: NaCl (100 mM), Tris-HCl (50 mM), EDTA (1 mM), MgCl_2_ (3 mM), protease inhibitor (1 μL/mL), pH 7.4. Next, 100 μL of the protein suspension was mixed with 200 μL of assay buffer containing adenosine deaminase (Sigma-Aldrich; final concentration, 3 U/mL), guanosine 5′-diphosphate (Abcam; 100 μM), and either the A_2A_R-selective antagonist, SCH58261 (Tocris; 1 μM) to measure non-specific binding or its vehicle, DMSO (0.1% v/v) to yield the total binding. This mixture also contained the A_2A_R-selective radioligand ^3^H-ZM241385 (American Radiolabeled Chemicals, St. Louis, MO, USA; specific activity, 30 mCi/mmol) at a final concentration of 2.63 nM. The binding assay was carried out in duplicate. The remaining 50 μL of protein aliquots were used to determine protein concentration with the BCA method. The mixtures (containing 20.6 ± 1.4 μg of protein) were left to incubate for 2 h at room temperature in Eppendorf-tubes, then were rapidly transferred into 15 mL of ice-cold washing solution (Tris-HCl, 50 mM, BSA 0.1% v/w), and instantly vacuum-filtered with the help of a Millipore filtration unit, containing Whatman GF/B glass microfiber filters, which had been soaked overnight in Tris-HCl (10 mM), pH 9.1, containing 0.25% v/v of the cationic polymer polyethylenimine (Sigma; Bruns et al., 1983). The glass tubes were rinsed with an additional 15 mL of washing solution onto the filters. The filters then were harvested into 3 mL of Aquasafe scintillation liquid and after 24 h, were counted for tritium with the help of a Tricarb β-counter (PerkinElmer). Binding values are expressed as fmol binding sites per mg protein.

### Behavioral Experiments

Behavioral analyses started 21 days post-surgery and were conducted between 8:00 AM and 6:00 PM under a low intensity red light (12 lx), after habituation of the animals to the room for at least 1 h and with care to clean all apparatus with ethanol after testing each animal to eliminate olfactory cues. We carried out two groups of experiments, all video-taped and analyzed using the ANY-maze video tracking system (Stoelting, Wood Dale, IL, USA).

### Experimental Set I

The first group of rats were sequentially exposed to the following behavioral tests with a 24 h interval in between them: the elevated plus maze, in order to assess anxiety-like behavior; the open field test to assess locomotor activity as well as anxiety-like behavior; and the splash test in order to evaluate mood alterations.

The elevated plus maze was carried out in an elevated plus-shaped maze with two open arms arranged perpendicularly to two closed arms, as previously described (Kaster et al., 2015). Rats were allowed to explore the maze for 10 min. The general principle of this test is that more “anxious” animals will likely explore less the risky open arms as opposed to the closed arms, which are perceived as safer. Thus, anxiety-like behavior was measured as a lower percentage of open arm entries (Pellow et al., 1985). Entries were counted whenever all the four paws of the animal crossed into one of the arms. The open field test was carried out in a square-shaped arena (1 × 1 m) with defined peripheral and central (36% of total area) zones. Rats were allowed to explore the arena for 10 min and only the first 5 min were analyzed (Gonçalves et al., 2015). Locomotor activity was measured as the total distance traveled and anxiety-like behavior was measured by the time spent in the center zone of the arena, which is perceived as a more threatening area (Choleris et al., 2001; Prut and Belzung, 2003).

The splash test was used as a measure of anxiety- and depressive-like behavior as previously described (Kaster et al., 2015). We measured grooming bouts (head washing and nose/face and body grooming) over 5 min after a 10% sucrose solution was splashed on the dorsal coat of the animal (Yalcin et al., 2005).

### Experimental Set II

This second set of experiments sequentially tested rats in a delay-based cost-benefit decision making paradigm in a T-maze, followed by spatial working memory tests using a Y-maze and a repeated trial Morris water maze (MWM).

### Delay-Based Cost-Benefit Decision-Making Paradigm in a T-maze

This test is based on delay aversion, which is used as a measure of impulsive decision making or impulsive choice (Pattij and Vanderschuren, 2008). Animals had to choose between a large-but-delayed and small-but-immediate reward (adapted from Bizot et al., 2007). The testing apparatus was a gray-colored T-maze, built out of PVC, with 50-cm-high walls, consisting of a starting runway, ending in two perpendicular 50-cm-long, 15-cm-wide arms. Four removable guillotine wood doors were vertically inserted at the entry and 10-cm from the end of each arm. The space between doors in each arm was enough to accommodate a rat. One week before starting the behavioral tests, rats were food-restricted to achieve 90% of their original weight. During that time, palatable dog chow pellets (Royal Canin Junior^®^) were given to the rats to habituate them to the new food. The task was divided into three different phases: habituation, training and testing phase.

#### Habituation

Rats were individually placed on the starting runway and allowed to freely explore the apparatus. Each arm had three pellets, including the starting runway. After 5 min, the number of pellets ingested was verified. If an animal had not eaten all the pellets, it was subjected to a new habituation trial. Up to five trials were conducted each day. After eating all the pellets, the rats progressed to the training phase. The number of habituation trials to reach training criterion was recorded.

#### Training Phase

Rats were run in the maze where one arm of the apparatus had a small reward (0.5 pellet) and the other had a large (two pellets) reward. The arm where the large reward was placed was randomly selected for each rat, but it was always on the same side throughout the experiment for a given rat. Rats were individually placed on the starting runway and had equal access to both arms. Both doors in the chosen arm were opened when the animal turned to its direction. As soon as the first door was crossed, it was closed to prevent the rat from escaping. The second door in that arm remained open to allow the animal to eat the reward. Then, another trial was carried out until a session of five trials was complete. Up to two sessions of five trials were conducted in the same day. The criterion to progress to the testing phase was choosing the large reward at least four times in five trials in two consecutive sessions. Otherwise, further trials were carried out in the next day. The number of training sessions to reach testing criterion was recorded.

#### Testing Phase

The test was conducted in five consecutive days, each day consisting of five trials. A delay of 15 s was imposed before the rat had access to the large reward, i.e., after choosing the arm with the large reward, both doors were closed right after the animal crossed the first one, keeping the animal between doors during this period. No delay was imposed after entering the small reward arm. The number of choices of the large reward was recorded for each day.

### Working Memory Tests

The Y-maze spontaneous alternation test was carried out as previously described (Augusto et al., 2013). The rats explored the maze for 8 min. The spontaneous alternation test takes advantage of the natural tendency of animals to choose a different arm than the one previously chosen (Dudchenko, 2004). In a correct sequence, a rat chooses a different arm in each of the successive three entries. The percentage of alternation in correct sequences was used to evaluate spatial working memory.

The repeated trial MWM was carried out in a circular pool (100 cm in diameter, 55 cm high), filled with water at 26°C. A platform (10 cm in diameter) was placed just under the surface of the water. The extra-maze cues in the testing room were kept constant. The test was adapted from a four-trial repeated acquisition protocol described in previous studies (Whishaw, 1985; Zhou et al., 2009) with four consecutive daily trials repeated during four consecutive days. The interval between trials was less than 1 min. The platform was moved to a new quadrant every day, but kept in the same position for all trials on the same day. The rats were allowed to swim until they reach the platform. Working memory was evaluated through the latency of escape from the starting point to the platform.

### Statistical Analysis

Statistical analyses were performed using Prism 6 GraphPad Software. Data are expressed as mean ± standard error of the mean (SEM). Data were analyzed using unpaired Student’s *t*-test and two-way ANOVA for repeated measures, followed by Bonferroni *post hoc* test as appropriate. *p* < 0.05 was taken as statistically significant.

## Results

**Figure 1:**
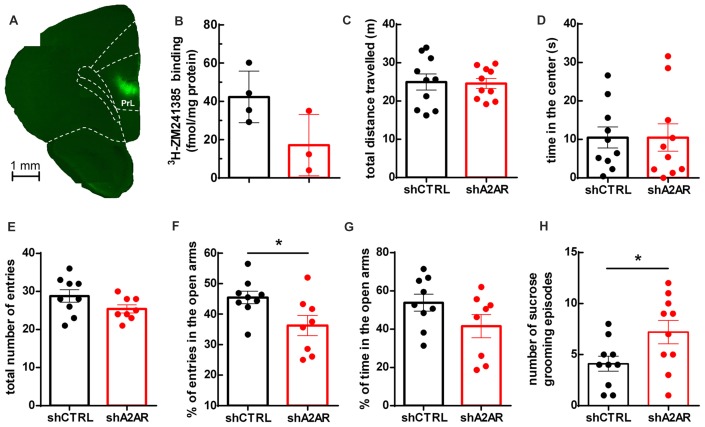
Effect of downregulatingadenosine A_2A_ receptors (A_2A_R) in the prelimbic medial prefrontal cortex (PLmPFC) on locomotion and anxiety- and depressive-like behavior. **(A)** Lentivector constructs containing small hairpin RNA targeting A_2A_R (shA_2A_R) together with enhanced green fluorescent protein (EGFP) reporter gene was effectively transduced in the PLmPFC, as shown by EGFP labeling in the region. **(B)** Specific binding of the selective A_2A_R antagonist ^3^H-ZM241385 shows a decrease in A_2A_R protein density in shA_2A_R- as compared to shCTRL-treated rats (*n* = 3–4, *p* = 0.07). **(C)** The analysis of the performance in the open field test suggests no alteration of exploratory activity, since the total distance traveled was similar between shA_2A_R- and shCTRL-treated rats. **(D)** There was also no change in the time spent in the center of the open field between shA_2A_R- and shCTRL-treated rats. **(E)** In the elevated plus maze there was no change in the total number of arm entries. **(F)** The performance in the elevated plus maze test suggests a mild anxiogenic effect resulting from the downregulation of PLmPFC A_2A_R, since shA_2A_R-treated rats entered less in the open arms when compared with shCTRL-treated rats. **(G)** Time spent in the open arms was not significantly affected (*p* = 0.1173). **(H)** Likewise, the performance in the splash test (with 10% sucrose solution) revealed an increased number of sucrose grooming episodes in shA_2A_R- compared with shCTRL-treated rats. Behavioral data are mean ± SEM of 9–10 rats per group; *p < 0.05, unpaired Student’s *t*-test.

We generated lentivectors, with neuronal tropism (Lundberg et al., 2008), encoding shRNAs to selectively neutralize A_2A_R (shA_2A_R) together with EGFP. These lentivectors were injected into the PLmPFC (Figure 1A) of rats. Upon dissection of the PLmPFC 5 weeks post-injection, we performed radioligand biding assay with ^3^H-ZM241385, an A_2A_R ligand, to assess A_2A_R density. The density of A_2A_R in PLmPFC total membranes was 42.28 ± 13.46 fmol/mg protein for shCTRL-treated rats (*n* = 4) and 17.13 ± 16.03 fmol/mg protein in PLmPFC total membranes from shA_2A_R-treated rats Figure 1B), representing a 59 ± 18% decrease in A_2A_R protein density as compared to shCTRL-treated rats (*n* = 3–4), a down-regulation similar to that achieved in the striatum (Viana da Silva et al., 2016).

### Downregulation of A_2A_R in the PLmPFC Induces Slight Mood Alterations With No Changes of Locomotor Activity

We then evaluated if the downregulation of A_2A_R in the PLmPFC impacted on locomotor activity. As shown in Figure 1C, there was no difference in the total distance traveled between shA_2A_R- and shCTRL-treated rats (24.59 ± 1.28 m for shA_2A_R-treated rats vs. 24.96 ± 2.10 m for shCTRL-treated rats, *n* = 10, *p* = 0.8830), suggesting that the exploratory behavior was not affected. Regarding anxiety-like behavior, the results were less clear: there was no difference in the time spent in the center of the open field between shA_2A_R- and shCTRL-treated rats (10.48 ± 3.58 s for shA_2A_R-treated rats vs. 10.49 ± 2.72 s for shCTRL-treated rats, *n* = 10, *p* = 0.9983; Figure 1D). However, in the elevated plus maze test, while total number of arm entries remained unchanged (25.38 ± 1.07 *n* = 8, for shA_2A_R-treated rats vs. 28.78 ± 1.64, *n* = 9, for shCTRL-treated, *p* = 0.1116; Figure 1E), shA_2A_R-treated rats entered less in the open arms as compared with shCTRL-treated rats (36.24 ± 3.32%, *n* = 8, for shA_2A_R-treated rats vs. 53.86 ± 4.46%, *n* = 9, for shCTRL-treated, *p* = 0.0289; Figure 1F). In contrast, the time spent in the open arms was not significantly altered (41.56 ± 6.04% *n* = 8, for shA_2AR_-treated rats vs. 53.86 ± 4.46%, *n* = 9, for shCTRL-treated, *p* = 0.1173; Figure 1G). In the splash test, there was an increase in sucrose grooming frequency (7.20 ± 1.14 events for shA_2A_R-treated rats vs. 4.10 ± 0.737 events for shCTRL-treated rats, *n* = 10, *p* = 0.0351; Figure 1H). Altogether, these data suggest that downregulating PLmPFC A_2A_R might result in a discrete anxiogenic profile.

### Downregulation of A_2A_R in the PLmPFC Renders Rats More Averse to Delay

**Figure 2:**
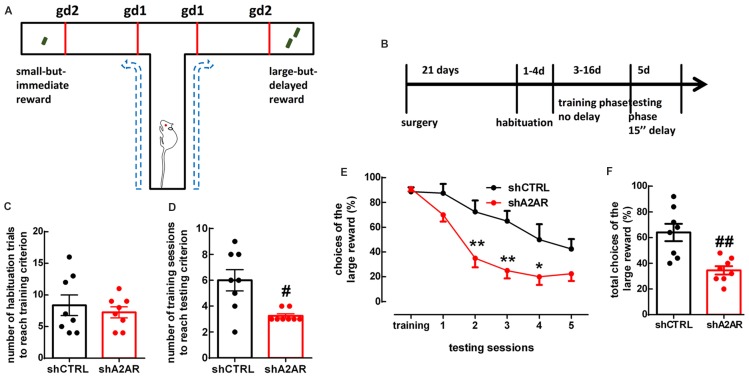
Downregulation of A_2A_R in the PLmPFC increases impulsive choice. **(A)** Scheme of the testing apparatus (gd, guillotine door). **(B)** Scheme of the experimental design. **(C)** shA_2A_R- and shCTRL-treated rats required a similar number of habituation trials to reach training criterion. **(D)** shA_2A_R-treated rats required less training sessions than shCTRL-treated rats to reach testing criterion (when they learn to choose the large reward); **(E,F)** with a 15-s-imposed delay, shA_2A_R-treated rats were more intolerant to delay as compared to shCTRL-treated rats, as shown by their decreased choice of the large reward. Data are mean ± SEM. ^#^*p* < 0.05 and ^##^*p* < 0.01, unpaired Student’s *t*-test; *p < 0.05 and **p < 0.01, two-way analysis of variance (ANOVA) for repeated measures, followed by Bonferroni *post hoc* test.

We used a delay-based cost-benefit decision making paradigm in a T-maze (Figures 2A,B) to evaluate preference for a small immediate reward over a larger, but delayed reward. In the habituation phase, shA_2A_R- and shCTRL-treated rats needed a similar number of habituation trials to reach training criterion, i.e., they learned similarly that there was a reward at the end of two arms (shA_2A_R-treated rats learned over 7.25 ± 0.88 trials, whereas shCTRL-treated rats learned over 8.38 ± 1.64 trials, *n* = 8, *p* = 0.5575; Figure 2C). In the training phase, shA_2A_R-treated rats needed a lower number of training sessions to reach testing criterion, i.e., they learned faster to choose the larger reward as compared to shCTRL-treated rats (shA_2A_R-treated rats learned over 3.25 ± 0.16 sessions, whereas shCTRL-treated rats learned over 6.00 ± 0.82 sessions, *n* = 8, *p* = 0.0122; Figure 2D). However, in the testing phase, shA_2A_R-treated rats were more intolerant to a 15-s-imposed delay as compared to shCTRL-treated rats, suggesting an increase in impulsive choice upon down-regulation of A_2A_R in the PLmPFC. A repeated measures ANOVA analysis indicated a decrease in the choices of the large reward with increased number of sessions (*F*_(5,70)_ = 28.08, *p* < 0.0001), with shA_2A_R treatment (*F*_(1,14)_ = 13.64, *p* = 0.0024), and a session × shA_2A_R treatment interaction (*F*_(5,70)_ = 3.10, *p* = 0.0138; Figure 2E). The total number of choices of the large reward was 8.63 ± 0.82 in shA_2A_R-treated rats and 16.00 ± 1.67 in shCTRL-treated rats (*n* = 8, *p* = 0.0026; Figure 2F).

### Downregulation of A_2A_R in the PLmPFC Does Not Affect Spatial Working Memory

**Figure 3:**
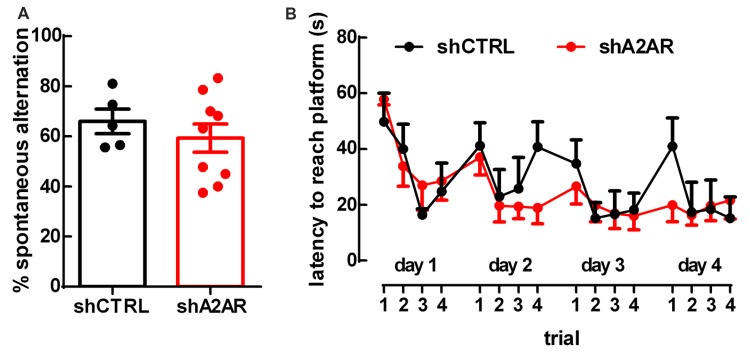
Downregulation of A_2A_R in the PLmPFC does not affect spatial working memory. **(A)** The performance in the Y-maze test revealed no change of the percentage of spontaneous alternation in shA_2A_R-compared to shCTRL-treated rats. **(B)** The repeated trial Morris water maze (MWM) paradigm revealed that shA_2A_R-treated rats had no changes in the latency to reach the platform as compared to shCTRL-treated rats. Data are mean ± SEM of *n* = 5 for shCTRL- and *n* = 9 for shA_2A_R-treated rats.

There were no differences in spontaneous alternation in the Y-maze test between shA_2A_R- and shCTRL-treated rats (65.87 ± 5.61%, *n* = 9, for shA_2A_R-treated rats vs. 66.44 ± 3.99%, *n* = 5, for shCTRL-treated rats, *p* = 0.9350; Figure 3A). In the MWM test, repeated measures ANOVA revealed an effect for trial (*F*_(15,180)_ = 4.63, *p* < 0.0001), but not for shA_2A_R treatment (*F*_(1,12)_ = 0.31, *p* = 0.5853; Figure 3B). Overall, these data suggest that A_2A_R in the PLmPFC have no impact on spatial working memory.

## Discussion

We have recently reported that A_2A_R in the PLmPFC control long-term plasticity of excitatory synaptic transmission onto fast spiking interneurons (Kerkhofs et al., 2018) and that they are necessary for dopamine-induced decrease in population activity (Real et al., 2018). Thus, we anticipated a role for PFC A_2A_R in the control of PFC-dependent behaviors, which include anxiety-like behavior (Calhoon and Tye, 2015; Tovote et al., 2015), cost-benefit decision-making (Bailey et al., 2016) and working memory (Goldman-Rakic, 1999; Fuster, 2001). The most evident effect resulting from the down-regulation of A_2A_R selectively in the PLmPFC was increased aversion to delay, suggestive of increased impulsive decision making, while only a discrete increase in anxiety-like behavior was observed, and spatial working memory was not significantly affected.

A_2A_R, in particular those in the nucleus accumbens, have been consistently implicated in cost-benefit decision-making in which the cost is physical effort (e.g., Font et al., 2008; Mingote et al., 2008; Pardo et al., 2012; Nunes et al., 2013). Specifically, activation of A_2A_R decreased lever pressing for the preferred food as opposed to eating the readily available less preferred chow (Font et al., 2008) and disrupted performance in an instrumental task with high work demands (Mingote et al., 2008), while A_2A_R blockade or genetic deletion attenuated haloperidol (dopamine D_2_ receptor antagonist)-induced decrease in the choice of the high reward arm of a T-maze that was accessible after climbing a barrier (Pardo et al., 2012). Now, we show that PLmPFC A_2A_R have the opposite effect on a cost-benefit decision-making task in which the cost was delay. Decisions about different, yet interrelated, types of costs have dissociable neural circuits and neurochemical mechanisms (Rudebeck et al., 2006; Floresco et al., 2008; Bailey et al., 2016), which may have contributed to this difference. Furthermore, it is known that A_2A_R are able to modulate the same behavior in opposite direction, depending on the brain region that is being manipulated. That is, for instance, the case for fear memory (Wei et al., 2014; Simões et al., 2016) and psychomotor activity (Shen et al., 2008). Thus, a more comprehensive study involving region-selective manipulations of A_2A_R will be necessary to dissect their role across different types of decision costs.

The impact of PFC A_2A_R on delay-based decision making could either be due to a control of: (i) aversion to the holding chamber before the reward; (ii) an altered goal-directed to habit-based strategy; (iii) an altered subjective value of the reward; (iv) an altered spatial memory encoding; and (v) impulsivity. This is compatible with the main involvement of the accumbens-PFC-amygdala circuitry (Floresco and Ghods-Sharifi, 2007; Hauber and Sommer, 2009) as well as of the dorsal hippocampus (Liu et al., 2016) in T-maze based analysis of effort-based decision making, and with the ability of the PFC to control aversive memories (Courtin et al., 2013), goal-directed behavior (Gourley and Taylor, 2016), the subjective value of rewards (Kable and Glimcher, 2007), processing of spatial memory (Jin and Maren, 2015) and impulsivity (Kim and Lee, 2011). However, the previous analysis of the role of A_2A_R in these different behaviors leads us to propose that PFC-A_2A_R mostly control delay-based decision making by controlling impulsivity. This contention stems from observations that: (i) A_2A_R control aversive memories, but this is fully accounted by the impact of amygdalar A_2A_R (Simões et al., 2016); (ii) A_2A_R control the shift from goal-directed to habit-based strategies, but this is fully accounted by the activity of A_2A_R in medial spiny neurons of different striatal regions (Yu et al., 2009; Li et al., 2016); (iii) A_2A_R control reward, but this is dependent on A_2A_R in the nucleus accumbens rather than PFC A_2A_R (Harper et al., 2006; Wydra et al., 2018); and (iv) A_2A_R control spatial memory, but this is fully accounted by A_2A_R in the dorsal hippocampus (Li P. et al., 2015; Pagnussat et al., 2015). Furthermore, we now report that PFC A_2A_R have a discrete impact on anxiety-like behaviors. Therefore, it is likely that the control by PFC A_2A_R of delay-based decision making might be a consequence of an ability of PFC A_2A_R to control impulsivity, which is often inferred from the analysis of delay-based decision tasks (Dalley et al., 2011; Kim and Lee, 2011). This contention that PFC A_2A_R might control delay-based decision making by controlling impulsivity is in agreement with the key role of the PFC in gating impulsivity (Sripada et al., 2011; Mason et al., 2014). However, it should be made clear that this is an indirect inference rather than a direct demonstration and future work should address if PFC A_2A_R also control others forms of impulsivity apart from impulsive intertemporal choice, such as impulsivity based on speed instead of accuracy (see Kim and Lee, 2011).

Our finding of increased delay aversion upon decreased function of PLmPFC A_2A_R seems to fully account for the exacerbation of waiting impulsivity observed upon systemic antagonism of A_2A_R (Oliveros et al., 2017). Likewise, there also seems to be a positive correlation between the intake of caffeine (Grant and Chamberlain, 2018) or caffeinated alcoholic beverages (Amlung et al., 2013; Heinz et al., 2013) and higher impulsivity. Although caffeine is a mixed antagonist of A_1_/A_2A_R antagonist (Fredholm et al., 2005), and its intake is already biased by the predisposition to impulsivity (Waldeck and Miller, 1997; Jones and Lejuez, 2005), animal studies indicate that caffeine can actually reduce impulsive choice behavior only in the sub-population of rodents with medium-to-high basal level of impulsivity (Barbelivien et al., 2008). Thus, blockade of adenosine receptors seems to work as a normalizer of function, bolstering impulsivity in low-impulsivity individuals and dampening impulsivity when it is already elevated. This putative shift of A_2A_R function may be associated with stressful conditions in the brain (reviewed in Cunha, 2016) or with a disbalance among the PFC, dorsal hippocampus and nucleus accumbens in the control of impulsivity (Kim and Lee, 2011; Monterosso et al., 2001), since synaptic plasticity is differently regulated by A_2A_R in each of these brain areas (D’Alcantara et al., 2001; Costenla et al., 2011; Kerkhofs et al., 2018). This possibility allows reconciling the presently observed increased impulsivity upon selective downregulation of A_2A_R in the PLmPFC with the beneficial effect of caffeine and A_2A_R antagonists in processes such as memory deterioration (Cunha and Agostinho, 2010; Chen, 2014), ADHD (Pandolfo et al., 2013), schizophrenia (Rial et al., 2014), effort-based decision-making (e.g., Pardo et al., 2012; Nunes et al., 2013), ethanol consumption (Nam et al., 2013) or psychomotor responses triggered by drugs of abuse (e.g., Shen et al., 2008; Matos et al., 2015), all of which are worsened with increased impulsivity.

The role of A_2A_R in the control of anxiety is not straightforward (reviewed in Cunha et al., 2008; Yamada et al., 2014). Accordingly, there was a discrete effect upon downregulating A_2A_R selectively in the PLmPFC on anxiety-like behavior in the elevated plus maze test and in the splash test, whereas no effect was observed in the open field test. Although previous human genetic association studies implicate polymorphisms of the A_2A_R gene in caffeine-induced anxiety (Alsene et al., 2003; Tsai et al., 2006), there is some discrepancy on the impact on anxiety-like behaviors of A_2A_R genetic deletions and A_2A_R pharmacological antagonism (e.g., Ledent et al., 1997; El Yacoubi et al., 2000; Kaster et al., 2015) as well as A_2A_R overexpression (Giménez-Llort et al., 2007; Coelho et al., 2014). The inconsistency in these global manipulations of A_2A_R might result from a differential contribution of A_2A_R in different brain regions. This is exemplified by the observations that the deletion of A_2A_R in striatal neurons does not affect anxiety-like behavior, while deletion of A_2A_R in the entire forebrain or focal deletion of hippocampal A_2A_R both produce an anxiolytic phenotype (Wei et al., 2014). Our results add further complexity to the A_2A_R-mediated modulation of anxious behavior and warrants future region-selective studies to unravel the impact of A_2A_R in different circuits in the control this behavior.

Given that the downregulation of A_2A_R in the PLmPFC resulted in a discrete anxious phenotype, caution must be taken when inferring impulsive choice behavior from a T-maze delay-based cost-benefit decision making analysis. The enclosure of animals with an anxious phenotype in a small compartment between two arms of the T-maze during the delay period could have induced a context aversion, leading the rats to choose the small reward solely as a result of a cost-benefit re-evaluation rather than impulsivity. Furthermore, the subjective value of the large food reward was greater in shA_2A_R- as compared to shCTRL-treated rats as they needed lower number of training sessions to reach testing criterion. Because of this, the extinction of this subjective value could also be faster, adding a confound to our observations. It is known that PLmPFC and A_2A_R regulate fear responses. However, the involvement of A_2A_R in fear responses is complex, as the nature of regulation depends on the manipulated brain region (Wei et al., 2014; Simões et al., 2016). If A_2A_R in the PLmPFC also regulates fear responses is not known. Interestingly, impulsive choice behavior has been shown to predict greater anxiety-like behavior in rats (Stein et al., 2015), and in humans, anxious individuals were shown to be impulsive decision-makers in the delay discounting task (Xia et al., 2017), both in agreement with our findings. Thus, future studies to clarify the role of A_2A_R in anxiety and fear responses and their relationship to impulsive behavior will be useful to dissociate between the impact of PLmPFC A_2A_R on impulsive decision making vs. on reward value- and context-dependent re-evaluation of cost-benefit during decision making.

The final PFC-related behavioral output that was investigated was working memory, which is bolstered upon pharmacological and genetic ablation of A_2A_R, both in physiological and pathological situations (reviewed in Chen, 2014), whereas transgenic overexpression of A_2A_R in the cortex of rats impairs working memory (Giménez-Llort et al., 2007). Working memory is a short-lasting on-line memory buffer system that holds behaviorally relevant information to ongoing tasks and relies on a network of brain regions connected to and orchestrated by the PFC (Goldman-Rakic, 1999; Fuster, 2001). However, we now show that the genetic downregulation of A_2A_R selectively in the PLmPFC of rats does not affect spatial working memory when assessed as the spontaneous alternation in the Y-maze and it has an inconsistent effect on working memory assessed in the repeated trial MWM test. Our findings are in line with a recent report that optogenetic activation of A_2A_R signaling pathways in the mPFC did not affect spatial working in the Y-maze test (Li et al., 2018); in contrast, the selective manipulation of A_2A_R in the striatum is sufficient to control working memory (Zhou et al., 2009; Wei et al., 2011) in a manner equivalent to the improvement of spatial working memory upon systemic antagonism of A_2A_R (Augusto et al., 2013; Li et al., 2018). Thus, it seems that striatal A_2A_R override mPFC A_2A_R in controlling working memory performance in physiological conditions. However, it remains to be determined to which extent PFC A_2A_R might contribute to the recovery of the deterioration of working memory performance afforded by A_2A_R blockade in different pathological conditions (Horita et al., 2013; Li W. et al., 2015).

Altogether, our findings that PLmPFC A_2A_R mediate impulsive choice constitutes the first direct demonstration of a role of A_2A_R in the control of behavior in physiological conditions. We have recently shown that PFC A_2A_R LTP in excitatory synapses onto fast spiking interneurons and control PLmPFC network activity (Kerkhofs et al., 2018). Thus, future research targeting selectively A_2A_R in PLmPFC fast spiking interneurons will be needed to clarify whether specifically A_2A_R located on glutamatergic synapses impinging on fast spiking interneurons control decision making and impulsive choice, or these behaviors are rather dependent on cooperation among A_2A_R located in different cell types. Furthermore, the differences observed between the selective manipulation of A_2A_R in the PLmPFC and more global alterations of A_2A_R function clearly warrant the need of future studies to dissect the hierarchy of the different roles of A_2A_R in different brain regions in the control of mood and cognition. Additionally, since decision making and impulsive choice is also modulated by dopamine receptors, it will also be interesting to probe whether the effect of A_2A_R on impulsive choice involves interaction with dopamine D_2_R, especially because antagonism and genetic deletion of A_2A_R dampen dopamine-mediated decrease in PFC network activity (Real et al., 2018). Finally, the up-regulation of A_2A_R in synapses upon brain disease condition (reviewed in Cunha, 2016), namely in the PFC (Pandolfo et al., 2013), heralds the potential of A_2A_R as relevant players controlling the pathophysiology of several neuropsychiatric disorders (Cunha et al., 2008), which still remains to be explored.

## Author Contributions

DL, PP, NG, RC and SF designed the research. DL, PP, NG, NM, CS, JR, HS, AK and SF performed the experiments and analyzed the data. SF wrote the first draft of the manuscript. All authors commented on the manuscript text.

## Conflict of Interest Statement

RC is a scientific consultant for the Institute for Scientific Information on Coffee. The remaining authors declare that the research was conducted in the absence of any commercial or financial relationships that could be construed as a potential conflict of interest.
